# Nitrogen-Containing Gas Sensing Properties of 2-D Ti_2_N and Its Derivative Nanosheets: Electronic Structures Insight

**DOI:** 10.3390/nano11092459

**Published:** 2021-09-21

**Authors:** Hongni Zhang, Wenzheng Du, Jianjun Zhang, Rajeev Ahuja, Zhao Qian

**Affiliations:** 1 Key Laboratory of Liquid-Solid Structural Evolution and Processing of Materials (Ministry of Education), Shandong University, Jinan 250061, China; nini810@126.com (H.Z.); 201813740@mail.sdu.edu.cn (W.D.); 15163625981@163.com (J.Z.); 2 College of Industry and Commerce, Shandong Management University, Jinan 250357, China; 3 Condensed Matter Theory, Department of Physics and Astronomy, Ångström Laboratory, Uppsala University, 75120 Uppsala, Sweden; rajeev.ahuja@physics.uu.se; 4 Department of Materials Science and Engineering, KTH Royal Institute of Technology, 10044 Stockholm, Sweden

**Keywords:** atomic structure, electronic structure, Density Functional Theory (DFT), MXenes, two-dimensional materials

## Abstract

In this work, the potentials of two-dimensional Ti_2_N and its derivative nanosheets Ti_2_NT_2_(T=O, F, OH) for some harmful nitrogen-containing gas (NCG) adsorption and sensing applications have been unveiled based on the quantum-mechanical Density Functional Theory calculations. It is found that the interactions between pure Ti_2_N and NCGs (including NO, NO_2_, and NH_3_ in this study) are very strong, in which NO and NO_2_ can even be dissociated, and this would poison the substrate of Ti_2_N monolayer and affect the stability of the sensing material. For the monolayer of Ti_2_NT_2_(T=O, F, OH) that is terminated by functional groups on surface, the adsorption energies of NCGs are greatly reduced, and a large amount of charges are transferred to the functional group, which is beneficial to the reversibility of the sensing material. The significant changes in work function imply the good sensitivity of the above mentioned materials. In addition, the fast response time further consolidates the prospect of two-dimensional Ti_2_NT_2_ as efficient NCGs’ sensing materials. This theoretical study would supply physical insight into the NCGs’ sensing mechanism of Ti_2_N based nanosheets and help experimentalists to design better 2-D materials for gas adsorption or sensing applications.

## 1. Introduction

Industrial pollutant emissions account for a large share of environmental pollution, and one of the most harmful is nitrogen-containing gas (NCGs), such as NO, NO_2_, and NH_3_. At the same time, the nitrogen-containing gases themselves are generally toxic and can damage the respiratory cells of humans and animals [1,2,3,4]. Therefore, there is an urgent need to explore efficient and alternative materials for the sensing or capturing of these gases. Although some current solid-state sensors can also detect toxic gases, there is still much room for improvement due to the increasingly high demand for sensitivity, selectivity, stability, and other factors [5,6]. Two-dimensional (2-D) materials have large contact areas with gas and special electronic properties; thus, their interactions with gas molecules have been widely studied, especially in gas sensing and capturing [7]. For example, Zhang et al. studied the adsorption behavior between four modified graphene substrates and small gas molecules (CO, NO, NO_2_, and NH_3_) and significantly improved the performance of the gas sensor by introducing doping or defects [8]. Liu et al. found that the GeSe monolayer had potential for NH_3_ gas capture and storage, as well as for the detection and catalysis of SO_2_ and NO_2_. The optical properties of this GeSe monolayer can also be tuned as a candidate material for optical sensors by adsorbing different small molecules [9]. Li et al. compared the structures and electronic properties of pure and two vacancy-defected MoS_2_ nanosheets with NO adsorption. Defects can effectively improve the NO-sensing performance [10].

Transition metal carbide or nitride MXenes are also one family of promising gas-sensing materials [11,12], where M is a transition metal and X is carbon or nitrogen. The general formula of MXenes is written as M_n_X_n−1_. During the preparation process in an aqueous solution containing fluoride ions, there may be some O, F, and OH functional groups formed [13,14,15]. This type of MXene with functional groups is written as M_n_X_n−1_T_n_(T=O, F, OH). MXenes are also widely reported in energy storage, electrode materials, ion batteries, and the spintronic and optical devices, due to their unique structures and properties [16,17,18,19,20,21,22,23,24].

After our review of previous literature, we found that some carbide-based MXenes have the potential to be used as sensors for molecules, such as amino acids and nucleobase [25,26]. With less exfoliation energy compared with other types, the synthesizability of the nitride MXenes is good [27], while there are fewer studies on their use in sensing NCGs [28,29,30,31,32]. In order to obtain a theoretical understanding of the nitride MXenes in terms of their sensing or capturing performance towards nitrogen-containing gas (NCGs), in this work, we conducted a detailed computational study on the adsorption behaviors of NCGs (including NO, NO_2_, and NH_3_) of Ti_2_N monolayer with bare surface and terminated with O, F, and OH functional groups. Density Functional Theory–based first-principles calculations were performed to unveil the adsorption structures, charge transfer, band structures, density of states, and work functions to stimulate and inspire the deep mechanism investigations.

## 2. Methods

DFT calculations were employed by using the projector augmented wave (PAW) [33] pseudo-potentials in a Vienna *Ab initio* Simulation Package (VASP) [34]. For the exchange-correlation functional, we used the Perdew-Burke-Ernzerhof (PBE) [35] form of the generalized gradient approximation (GGA). Grimme’s DFT-D2 method was used to describe the van der Waals interactions [36]. This method adds semi-empirical dispersion on the basis of conventional Kohn–Sham DFT energy. In calculations, Ti_2_N and Ti_2_NT_2_(T=O, F, OH) were constructed as 4 × 4 × 1 supercells, and a 20 Å vacuum layer was added in *Z*-axis direction to eliminate the interlayer interaction. The energy cutoff of 520 eV was used for wave functions’ expansion. The Brillouin zone integration was sampled with 5 × 5 **k**-grid mesh for geometry optimizations and 15 × 15 **k**-grid mesh for electronic properties calculations to achieve high accuracy. Geometry optimizations were performed by using the conjugated gradient method, and the convergence criterion was set to be 0.02 eV/Å on force and 1 × 10^−5^ eV/atom on energy. The adsorption energy (E_ads_) of nitrogen-containing gas (NCGs) on Ti_2_N or Ti_2_NT_2_(T=O, F, OH) is defined as follows:

E_ads_ = E_MXene + NCGs_ − E _NCGs_ − E_MXene_ (1)

where E_ads_, E_MXene + NCGs_, E_MXene_, and E_NCGs_ stand for the adsorption energy, total energies of optimized Ti_2_N or Ti_2_NT_2_(T=O, F, OH) MXene with adsorbed NCGs molecules, energies of pure MXene, and isolated NCGs (NO, NO_2_, and NH_3_) molecules respectively.

## 3. Results and Discussion

**Figure 1:**
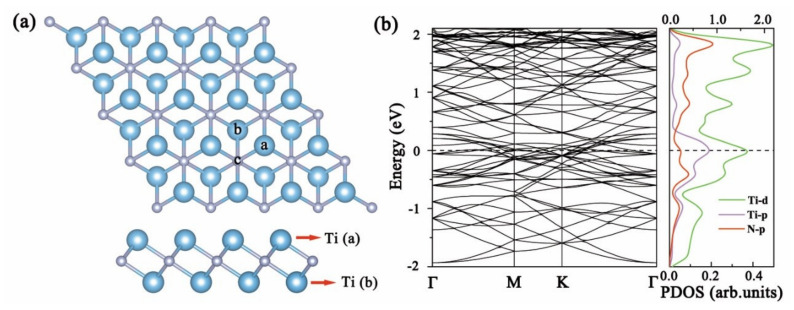
(**a**) Top and side view of the optimized structures for Ti_2_N monolayer (blue and silver balls represent Ti and N atoms, respectively). (**b**) Band structure and projected density of states of Ti_2_N monolayer (Ti*d*-orbital uses top abscissa and other orbitals use bottom coordinate).

First of all, the Ti_2_N MXene has a hexagonal structure with three layers of atoms: the middle is N-atoms layer; and the upper (a) and lower (b) layers are Ti atoms, as is shown in Figure 1a. After structural optimizations, the Ti-N bond length is 2.07 Å, the bond angle of the same layer Ti and N (Ti–N–Ti) and N–Ti–N are both 92.4°, and the bond angle of different-layer Ti and N (Ti–N–Ti) is 87.6°. The lattice parameter of the 4 × 4 supercell is a = b = 11.98 Å. These parameters are very consistent with the results of Ti_2_N studied in other works in the literature [37,38], indicating the reliability of the calculation results. The band structure and PDOS of the material are shown in Figure 1b; it can be seen that there are many electronic states available for occupation at the Fermi level, and Ti_2_N exhibits metallic properties. The states near the Fermi level are occupied by Ti electrons, where N’s contribution is relatively small, meaning that low-energy carriers mainly originate from Ti.

**Figure 2:**
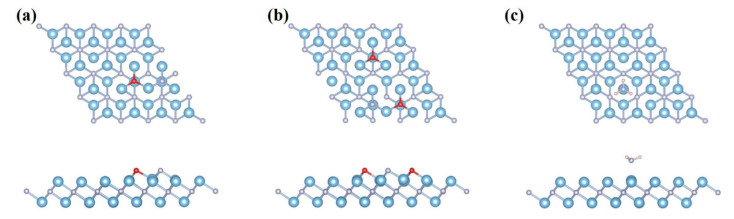
Top and side view of the optimized atomic structures for Ti_2_N monolayer after adsorbing (**a**) NO, (**b**) NO_2_, and (**c**) NH_3_ (blue, silver, red, and pink balls represent Ti, N, O, and H atoms, respectively).

**Figure 3:**
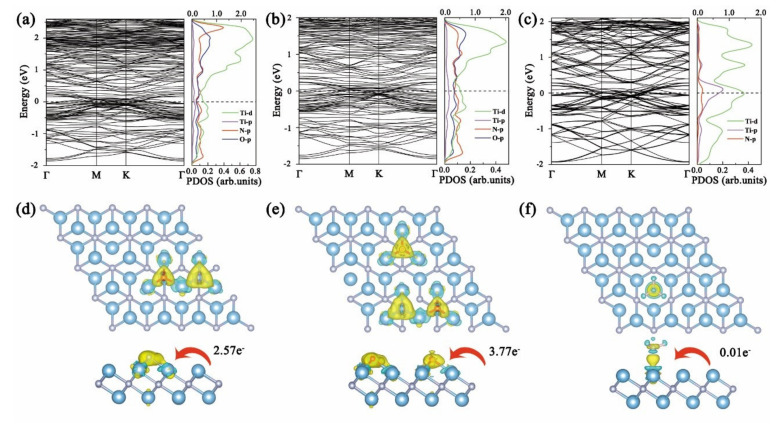
Band structures, PDOS and differential charge densities of Ti_2_N after adsorbing (**a**,**d**) NO, (**b**,**e**) NO_2_, and (**c**,**f**) NH_3_ (Ti*d*-orbital uses top abscissa and other orbitals use bottom coordinate; isovalue of NO and NO_2_ is set to 0.01 e/Å^3^, NH_3_ is set to 0.005 e/Å^3^).

In order to study the most stable adsorption configurations of NCGs on Ti_2_N MXene, the NO, NO_2_, and NH_3_ molecules were respectively placed at three different adsorption sites, namely on top of the Ti(a) atom shown in Figure 1a (site “a”), on top of the hollow Ti(b) atom (site “b”), or on top of the N atom (site “c”). The screened most stable configurations after adsorbing NCGs are shown in Figure 2. Both NO and NO_2_ show dissociative adsorption. The adsorption energy is far greater than those of other two-dimensional materials [39,40,41,42,43,44]. When NO is adsorbed on Ti_2_N, N and O atoms are adsorbed at the adjacent site “b” respectively. The distance between N and O is 3.07 Å, which is much larger than 1.15 Å of free NO gas molecule, indicating that the N–O bond is broken and NO dissociates. The dissociated N and O atoms form new bonds with Ti atoms in the upper layer of Ti_2_N, with bond lengths of 1.93 and 1.97 Å, respectively. Ti atoms protrude slightly upwards from its binding, and the Ti–N bond length on the surrounding matrix increases by about 0.14 Å. It can be seen from Figure 3a that, after dissociation, the N 2*p* and O 2*p* in NO and the nearby Ti 3*d*-orbitals have similar peak positions and obvious *p-d* hybridization in PDOS diagram. There is a strong bonding between NO and Ti_2_N, and the adsorption energy is as high as −9.809 eV. At the same time, the band structure shows that the dissociative adsorption of NO on Ti_2_N does not change the original metallic properties.

When NO_2_ interacts with Ti_2_N, it is interesting that NO_2_will also dissociate similar to NO adsorption, and all the N and O atoms are adsorbed at the site “b”. One O-atom keeps the same shape adsorbed at adjacent position, while the another O-atom is adsorbed away from it. The N-O bond length in NO_2_is increased to 3.07 Å. The dissociative adsorption energy is up to −14.336 eV. As can be seen from the electronic structure in Figure 3b, the *p-d* hybridization of N 2*p*-, O 2*p*-, and Ti 3*d*-orbitals corresponds with the strong bonding of Ti-N and Ti-O, respectively. The metallic behavior of Ti_2_N remains after NO_2_ dissociative adsorption. From the differential charge density in Figure 3d–f, it can be seen that the dissociation of NO and NO_2_ is caused by the excessive charge transfer from Ti_2_N MXene to the gas molecule. The Bader charge analysis shows that 2.57 e^−^ and 3.77 e^−^ charges were transferred from Ti_2_N to NO and NO_2_, respectively. The bulk of these transferred charges comes from the surrounding Ti atoms, and this is also the fundamental reason why the N-O bond is broken.

The adsorption of NH_3_ is completely different from those of NO and NO_2_. It is bound by associative adsorption with the binding distance of 2.2 Å and the E_ads_ value of−1.286 eV. As shown in Figure 3c, NH_3_ tends to be adsorbed at site “a” with N-atom below and three H-atoms evenly distributed above. The distance between the Ti atom at site “a” and the surrounding N atom is elongated to 2.14 Å, and the N–Ti–N bond angle is reduced to 88.3°. To figure out the binding and charge transfer mechanism between NH_3_ molecule and Ti_2_N monolayer, the band structure and PDOS were analyzed. It is difficult to find a pronounced overlap between N *p* and Ti*d* close to the Fermi level in Figure 3c. The amount of charge transfer is only 0.01 e^−^, which is far less than those of NO and NO_2_. The bonding between NH_3_ and Ti_2_N is very weak, and the band structure that is almost the same as the bare Ti_2_N monolayer also confirms this.

For ideal gas sensing application, excessive adsorption energy is obviously not feasible. The dissociative adsorption of NO and NO_2_ on Ti_2_N monolayer will obviously poison the substrate and make it fail to work cyclically. The dissociation of gas molecules results from the strong interaction between the bare Ti_2_N surface and O atoms. Thus, the pure Ti_2_N monolayer can be used as a kind of gas-trapping material instead of highly efficient sensor for NO and NO_2_. Compared with those two gases, the adsorption effect of NH_3_ is satisfactory. The associative adsorption with lower energy will not cause chemical poisoning, which is beneficial to Ti_2_N as a promising candidate material for reversible NH_3_ sensors. In actual preparation process, some functional groups are usually introduced onto the Ti_2_N monolayer, and these functional groups may have significant impacts on adsorption properties of the material. Considering this reality, in the following part, we recount how we studied the adsorption behaviors of the Ti_2_N monolayer terminated by O, F, and OH towards NCGs.

**Figure 4:**
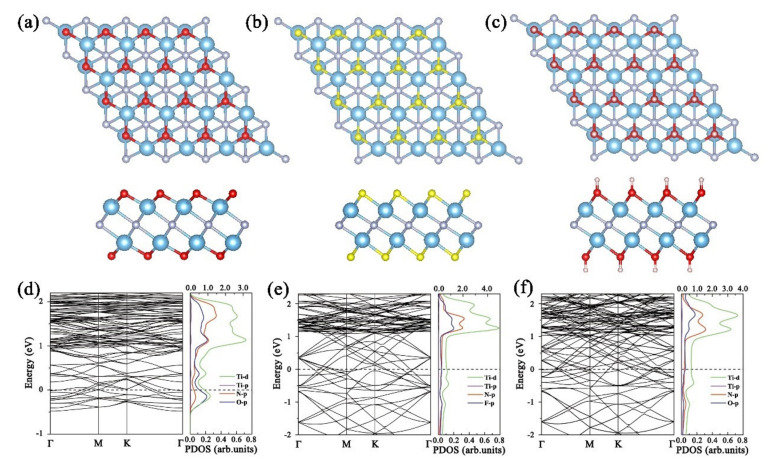
Top and side view of the optimized structures, band structures, and PDOS of (**a**,**d**) Ti_2_NO_2_, (**b**,**e**) Ti_2_NF_2_, and (**c**,**f**) Ti_2_N(OH)_2_ monolayers (blue, silver, red, pink, and yellow balls represent Ti, N, O, H, and F atoms, respectively. Ti*d*-orbital uses top abscissa and other orbitals use bottom coordinate).

We introduce O, F, and OH functional groups at three adsorption positions (site “a”, “b”, and “c”) of Ti_2_N, of which OH only considers the vertical direction (O atom is close to the matrix). All the functional groups tend to adsorb at site “b” with the lowest energy, as shown in Figure 4a–c. After adsorbing different functional groups, the Ti–N bonds show different degrees of extension: 2.164, 2.052, and 2.065 Å for Ti_2_NO_2_,Ti_2_NF_2_, and Ti_2_N(OH)_2_, respectively, which is due to the bonding between Ti and functional groups. Gouveia et al. reported possible atomic-layer-stacking-phase transitions of some MXenes [41]; thus, we also carefully checked this point in our research. It is found that the material system maintains phase stability and stacking way after introduction of those functional groups. The Bader charge analysis shows that 1.1, 0.8, and 0.8 e^−^ are respectively transferred to O, F, and OH from Ti_2_N. It can also be observed in Figure 4d–f that there is a strong overlap between the Ti*d*-orbital and the T (O, F, or OH)*d*-orbitals.

**Figure 5:**
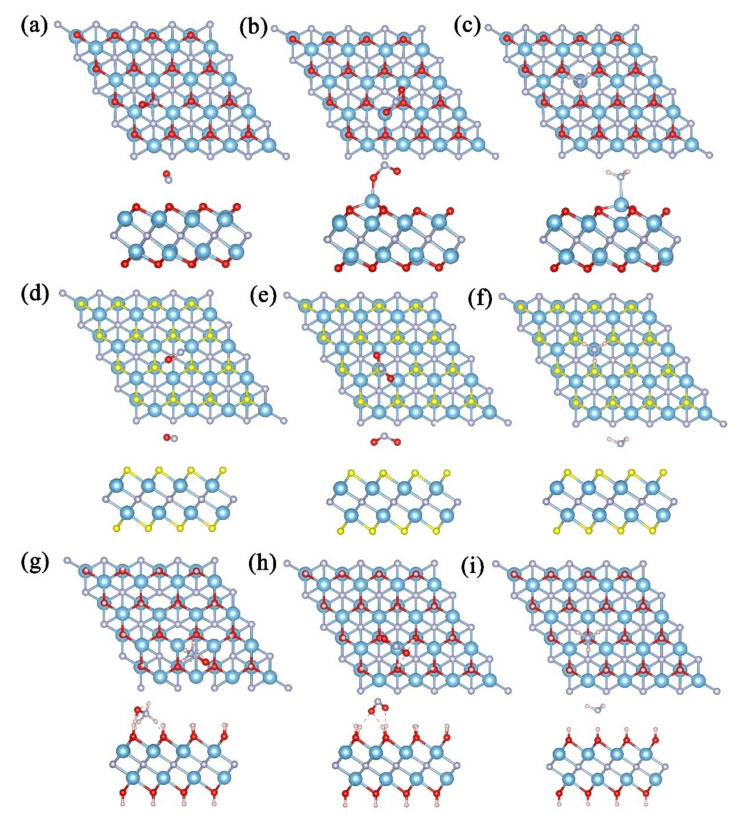
Top and side view of the optimized structures for Ti_2_NO_2_ after adsorbing (**a**) NO, (**b**) NO_2_, and (**c**) NH_3_; Ti_2_NF_2_ after adsorbing (**d**) NO, (**e**) NO_2_, and (**f**) NH_3_;and Ti_2_N(OH)_2_ after adsorbing (**g**) NO, (**h**) NO_2_, and (**i**) NH_3_ (blue, silver, red, pink, and yellow balls represent Ti, N, O, H, and F atoms, respectively).

After obtaining the stable configurations of Ti_2_NT_2_(T=O, F, OH), we placed various NCGs molecules at different adsorption sites (site “a”, “b”, and “c”), such as those in pure Ti_2_N. It can be seen from the most stable adsorption structures after optimizations in Figure 5 that all NCGs have no dissociative adsorption on Ti_2_NT_2_(T=O, F, OH). The binding distance is defined as the shortest direct distance between the NCGs molecule and the matrix; hereby, the binding distances of NO, NO_2_, and NH_3_ on Ti_2_NO_2_ are 2.082, 1.824, and 2.172 Å, respectively. The bond lengths of NO and NH_3_ after adsorption are the same as those of free gas molecules, while one of the N–O bonds of NO_2_ is stretched to 1.530 Å. The 2-D Ti_2_NO_2_ does not change significantly under NO environment, while the Ti atoms exposed to the adsorption positions of NO_2_ and NH_3_ are obviously dragged out. For NCGs adsorption on the surface of Ti_2_NF_2_, the molecular bond lengths and the matrix are almost unchanged with the adsorption distances of 2.951, 2.800, and 2.947 Å, respectively. It is surprising when NO is adsorbed on Ti_2_N(OH)_2_. The H atoms on the substrate bind with NO, where three H atoms combine with the N atom and one H atom combines with the O atom to form a hydroxyl lammonium-like product. The adsorption distances of NO_2_ and NH_3_are 1.625 and 1.702 Å, which are measured from the nearest H atom; thus, they are shorter than those of other Ti_2_NT_2_(T=O, F). While, the adsorption is not strong, especially the NH_3_ molecule has basically no change compared with the free molecule.

We can clearly see that the absolute values of adsorption energy of NCGs on Ti_2_NT_2_(T=O, F, OH) decrease sharply compared with the pure substrate in Table 1, especially on the surface of Ti_2_NF_2_. Since the H atoms of the OH functional groups fall off the Ti_2_N(OH)_2_ substrate and bind with NO, their decrease is not that large. On the pure Ti_2_N monolayer, a large amount of charge is transferred from Ti_2_N to NO and NO_2_. After the introduction of the functional group O, F, or OH, most of the charge transfer from Ti_2_N is captured by these functional groups, which leaves a very small charge amount for exchange with NCGs. In addition, one can see that the difference in adsorption energy of each NCG on substrate is obvious, indicating that the selectivity is good. As mentioned above, NO and NO_2_ are dissociatively adsorbed on pure Ti_2_N, which results in the poisoning of the Ti_2_N surface. Similarly, NO binds with H atoms of Ti_2_N(OH)_2_ and destroys the surface integrity of the substrate, which is detrimental to the sensing material. Thus, Ti_2_N(OH)_2_ is not suitable for NO sensing. From the perspective of adsorption energy, those of NO on Ti_2_NO_2_, NO_2_on Ti_2_NF_2_, and NH_3_on Ti_2_N(OH)_2_ are between physisorption and chemisorption, which are important to make sensors work reversibly and increase their efficiency [45].

**Figure 6:**
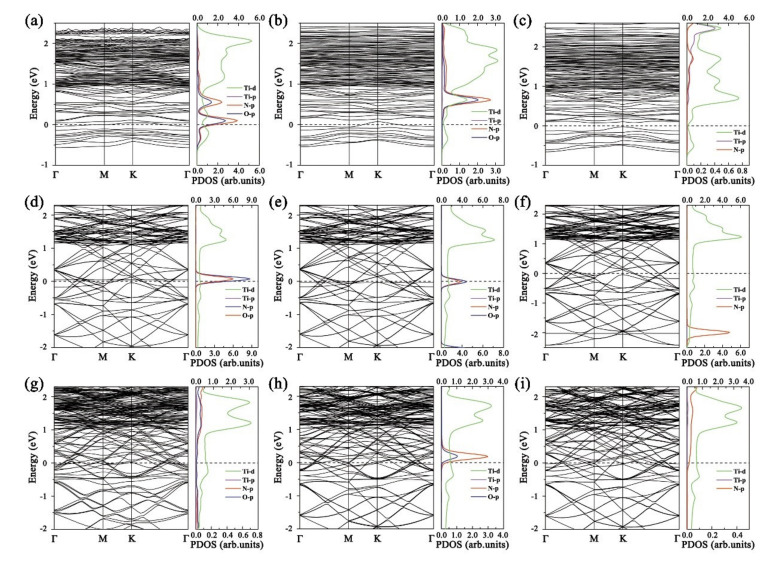
Band structures and PDOS of Ti_2_NO_2_ after adsorbing (**a**) NO, (**b**) NO_2_, and (**c**) NH_3_; Ti_2_NF_2_ after adsorbing (**d**) NO, (**e**) NO_2_, and (**f**) NH_3_; Ti_2_N(OH)_2_ after adsorbing (**g**) NO, (**h**) NO_2_, and (**i**) NH_3_ (Ti*d*-orbital uses top abscissa and other orbitals use bottom coordinate).

To better understand the effects of NCGs molecules on Ti_2_NT_2_(T=O, F, OH), the band structure and PDOS of all the adsorption systems are calculated and shown in Figure 6. Regardless of the type of NCGs adsorption, the system retains metallic properties. Only when Ti_2_NO_2_ is exposed to NO_2_, the Ti*d*-, O*p*-,and N *p*-orbitals show a certain degree of d-p hybridization, while the *p*- and *d*-orbitals in other PDOS do not overlap. When Ti_2_NO_2_ adsorbs NO, a flat band appears near the Fermi energy and indicates that the electrons are delocalized, which further proves that the interaction between NO and Ti_2_NO_2_ is not strong. This phenomenon is more obvious when NCGs are adsorbed on Ti_2_NF_2_ shown in Figure 6d–f. These results are consistent with the above discussion on energetics and charge-transfer profiles.

**Figure 7:**
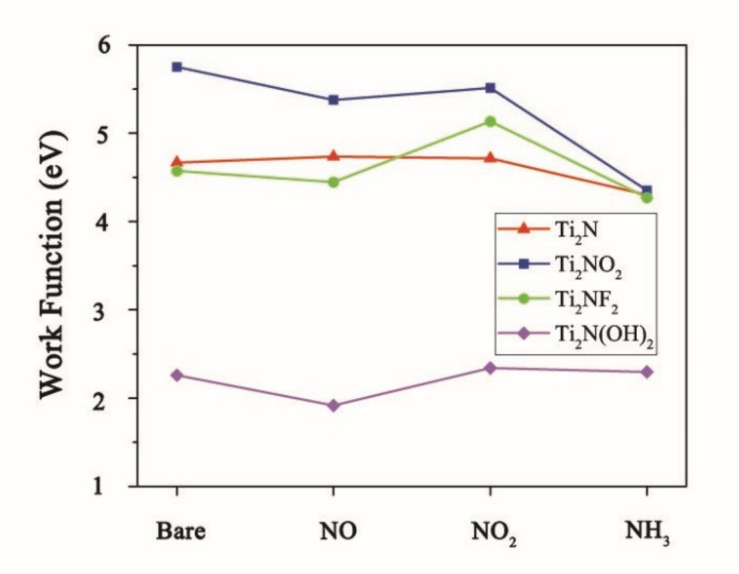
Work function change of Ti_2_N and Ti_2_NT_2_(T=O, F, OH) before and after adsorption of various NCGs.

An ideal sensing material not only needs to possess excellent gas-binding capability but also has a requirement for good sensitivity. The conductivity of materials, especially two-dimensional materials, is closely related with work function. During adsorption, if the electron affinity of gas molecule surpasses the work function of substrate materials, the molecule will grab electrons from the surface of materials and the molecule would be charged negatively; in contrast, the adsorbed molecule would be charged positively. Both two cases would change the electronic structure, as well as conductivity of materials. The work function [46] is defined as the least energy required to free an electron from the surface of a system, which can be calculated by the following equation:

φ= V_∞_ − E_f_ (2)

where φ, V_∞_, and E_f_ are the work function, electrostatic potential at the vacuum level, and the Fermi energy, respectively. Figure 7 shows the change of work function of Ti_2_N and Ti_2_NT_2_(T=O, F, OH) before and after adsorption of various NCGs. “Bare” represents the bare system (before adsorption), and the names of NCGs in abscissa represent various systems after adsorption of different gases. The work functions of Ti_2_NO_2_ and Ti_2_N(OH)_2_ decrease obviously after adsorbing NO, while that of Ti_2_NF_2_ increases significantly after adsorbing NO_2_. After adsorbing NH_3_, the work functions of Ti_2_N, Ti_2_NF_2_, and Ti_2_NO_2_ all decrease, and the change of Ti_2_NO_2_ is the most obvious. Because all the systems are metallic, even small changes in work function can cause large variation in conductivity [43]. Comprehensively considering the above results on adsorption structure and energy, Ti_2_NO_2_ can be used as a superior sensing candidate material for NO and NH_3_, and Ti_2_NF_2_ can be utilized as that for NO_2_. In addition, the potential of sensing properties of Ti_2_N, Ti_2_NF_2_, and Ti_2_N(OH)_2_ towards NH_3_ is also good.

**Figure 8:**
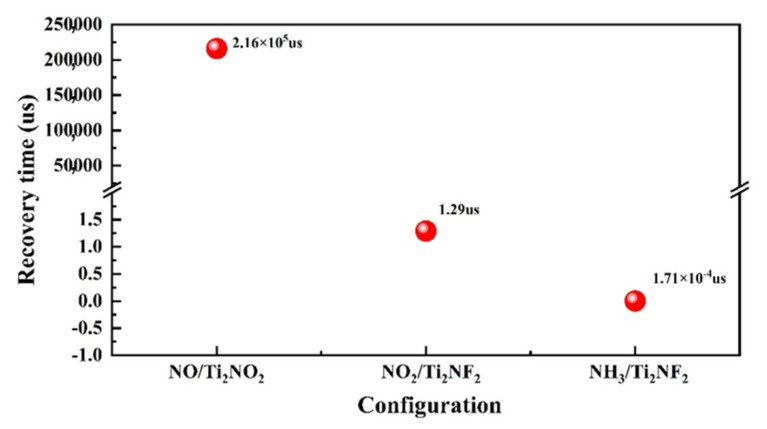
Recovery time of different sensing systems.

Finally, we discuss the recovery time (τ). An ideal gas-sensing material needs appropriate adsorption energy, and a shorter recovery time is better. To make a gas sensor work efficiently, a short recovery time is required. It can be calculated through transition state theory, namely by the following relationship [47,48]:

τ = υ^−1^exp(−E_ads_/kT) (3)

where υ, E_ads_, k, and T are the attempt frequency, adsorption energy, Boltzmann’s constant (8.62 × 10^−5^ eV K^−1^), and operational temperature, respectively. Under the same conditions, the increase in adsorption energy will lead to an exponential increase in recovery time; then the higher operating temperature compensation is required, which is detrimental to the sensor performance. In order to get a relatively reasonable value, we set the attempt frequency here to 10^12^s^−1^ based on traditional transition state theory [49,50,51]. The recovery time of NO on Ti_2_NO_2_ is calculated to be 2.16 × 10^5^ us, and that of NO_2_/Ti_2_NF_2_ and NH_3_/Ti_2_NF_2_ is 1.29 and 1.71 ×10^−4^ us, respectively. Here it can be seen in Figure 8 that the adsorption energy difference of less than 1 eV can be enlarged to several orders of magnitude in recovery time. Although NO on Ti_2_NO_2_ has the longest recovery time, the value is only 0.216 s. For the system of NH_3_/Ti_2_NF_2_, it has the shortest recovery time, which shows that the MXene system in this research can be promising. Therefore, when other conditions are met, the candidate materials with appropriate gas adsorption energy and short response time should be selected.

## 4. Summary and Outlook

In this work, we used the first-principles DFT calculations to investigate the 2-D Ti_2_N nanosheet and its Ti_2_NT_2_(T=O, F, OH) derivative materials for harmful nitrogen-containing gas (NGCs) adsorption and sensing applications. When NCGs are exposed on the surface of Ti_2_N, the dissociative adsorption of NO and NO_2_ occurs and destroys the stability of the nanosheet, which would make it hard to work reversibly, while NH_3_ does not have this effect on Ti_2_N. When studying the interaction mechanisms between NCGs and Ti_2_NT_2_(T=O, F, OH), it can be found that a large amount of charges accumulates at functional groups, thus greatly reducing the adsorption energy of NCGs in absolute value. The obvious change in work function, coupled with the metallic nature of the systems, would improve gas sensitivity. Appropriate gas adsorption energy can directly determine fast recovery time to ensure the efficiency and reversibility of sensors. This research gives the electronic structures origin and application prospect of Ti_2_NT_2_(T=O, F, OH) as efficient NCGs sensing materials and would inspire experimentalists to explore better 2-D candidates in the field.

## Figures and Tables

**Table 1:** Adsorption energy (E_ads_) and charge-transfer profiles of NCGs adsorption on 2-D Ti_2_N and Ti_2_NT_2_(T=O, F, OH).

NCGs	Adsorption Energy (eV)				Charge Transfer (e^−^)			
	Ti_2_N	Ti_2_NO_2_	Ti_2_NF_2_	Ti_2_N(OH)_2_	Ti_2_N	Ti_2_NO_2_	Ti_2_NF_2_	Ti_2_N(OH)_2_
NO	−9.81	−0.68	−0.15	−5.55	2.57	−0.24	−0.07	0.50
NO_2_	−14.34	−2.38	−0.36	−3.78	3.77	0.49	0.29	1.78
NH_3_	−1.29	−1.16	−0.13	−0.57	0.01	−0.18	−0.03	0.15

## Data Availability

Data are contained within the article.
